# Biomarkers in the Cerebrospinal Fluid of Patients with Psychotic Disorders Compared to Healthy Controls: A Systematic Review and Meta-Analysis

**DOI:** 10.1192/j.eurpsy.2022.328

**Published:** 2022-09-01

**Authors:** T. Rømer, R. Jeppesen, R. Christensen, M. Benros

**Affiliations:** University Hospital Copenhagen, Copenhagen Research Center For Mental Health (core), Hellerup, Denmark

**Keywords:** meta-analysis, PSYCHOTIC DISORDERS, cerebrospinal fluid, biomarkers

## Abstract

**Introduction:**

Biomarkers in CSF could provide etiological clues and diagnostic tools for psychotic disorders. However, an overview of all CSF findings in individuals with psychotic disorders compared to healthy controls is lacking.

**Objectives:**

To analyse CSF findings from individuals with psychotic disorders compared to healthy controls.

**Methods:**

PubMed, EMBASE, Cochrane Library, Web of Science, ClinicalTrials.gov, and PsycINFO were searched November 3rd, 2021. Case-control studies including patients with non-affective, psychotic disorder compared to healthy controls measuring at least one biomarker in CSF are included. Standardized Mean Differences (SMD) and random-effects analyses were used.

**Results:**

141 studies, covering 192 biomarkers, were included. 161 biomarkers have not previously been included in meta-analyses. Most markers measured showed no significant differences, including the dopamine metabolites HVA and DOPAC. Patients with psychotic disorders showed increased CSF levels of noradrenaline (SMD, 0.53; 95% CI, 0.15-0.90), MHPG (SMD, 0.30; 95% CI, 0.05-0.55), 5-HIAA (SMD, 0.11; 95 % CI, 0.01-0.21), kynurenic acid (SMD, 1.58; 95% CI, 0.26-2.91), kynurenine (SMD, 1.00; 95% CI, 0.58-1.42), IL-6 (SMD, 0.58; 95% CI, 0.39-0.77), IL-8 (SMD, 0.47; 95% CI, 0.18-0.77), anandamide (SMD, 0.78; 95% CI, 0.53-1.02), albumin ratio (SMD, 0.53; 95% CI, 0.10-0.96), total protein (SMD, 0.31; 95% CI, 0.14-0.48), and glucose (SMD, 0.57; 95% CI, 0.08-1.06). Neurotensin (SMD, -0.67; 95% CI, -0.89 to -0.46) and GABA (SMD, -0.29; 95% CI, -0.50 to -0.09) were decreased.

**Conclusions:**

These findings suggest that dysregulation of the immune and adrenergic system and blood-brain barrier dysfunction might play a role in the pathophysiology of psychotic disorders.

**Disclosure:**

No significant relationships.

